# Ubiquitin Reference Technique and Its Use in Ubiquitin-Lacking Prokaryotes

**DOI:** 10.1371/journal.pone.0067952

**Published:** 2013-06-25

**Authors:** Konstantin Piatkov, Emmanuelle Graciet, Alexander Varshavsky

**Affiliations:** 1 Division of Biology, California Institute of Technology, Pasadena, California, United States of America; 2 Smurfit Institute of Genetics, Trinity College Dublin, Ireland; University of North Dakota, United States of America

## Abstract

In a pulse-chase assay, the *in vivo* degradation of a protein is measured through a brief labeling of cells with, for example, a radioactive amino acid, followed by cessation of labeling and analysis of cell extracts prepared at different times afterward (“chase”), using immunoprecipitation, electrophoresis and autoradiography of a labeled protein of interest. A conventional pulse-chase assay is fraught with sources of data scatter, as the efficacy of labeling and immunoprecipitation can vary, and sample volumes can vary as well. The ubiquitin reference technique (URT), introduced in 1996, addresses these problems. In eukaryotes, a DNA-encoded linear fusion of ubiquitin to another protein is cleaved by deubiquitylases at the ubiquitin-protein junction. A URT assay uses a fusion in which the ubiquitin moiety is located between a downstream polypeptide (test protein) and an upstream polypeptide (a long-lived reference protein). The cotranslational cleavage of a URT fusion by deubiquitylases after the last residue of ubiquitin produces, at the initially equimolar ratio, a test protein with a desired N-terminal residue and a reference protein containing C-terminal ubiquitin moiety. In addition to being more accurate than pulse-chases without a reference, URT makes it possible to detect and measure the degradation of a test protein during the pulse (before the chase). Because prokaryotes, including Gram-negative bacteria such as, for example, *Escherichia coli* and *Vibrio vulnificus*, lack the ubiquitin system, the use of URT in such cells requires ectopic expression of a deubiquitylase. We describe designs and applications of plasmid vectors that coexpress, in bacteria, both a URT-type fusion and Ubp1, a deubiquitylase of the yeast *Saccharomyces cerevisiae*. This single-plasmid approach extends the accuracy-enhancing URT assay to studies of protein degradation in prokaryotes.

## Introduction

Ubiquitin (Ub) is a 76-residue eukaryotic protein that exists in cells either free or covalently linked to many different proteins, often in the form of poly-Ub chains. Ub-protein conjugation can signal either degradation of a ubiquitylated protein by the proteasome or other metabolic fates [1]–[3]. Natural Ub genes encode linear fusions of Ub either to itself (poly-Ub genes) or to other proteins. Such fusions are cotranslationally cleaved by deubiquitylases (DUBs), yielding mature Ub [1], [4]–[6].

A method based on properties of DUBs and termed the Ub fusion technique was introduced in 1986 [7]–[9]. In this approach, a segment of DNA encoding Ub is joined, in-frame, to DNA encoding a protein of interest, resulting in a Ub-X-protein fusion, with the amino acid X being a junctional residue that can be varied by site-directed mutagenesis. Expression of such a fusion in eukaryotes results in its cotranslational cleavage by DUBs at the Ub-X junction. A eukaryotic cell contains multiple DUBs that share the ability to recognize the Ub moiety and to cleave immediately after its C-terminal Gly76 residue [1], [10].

Because most DUBs can cleave a linear Ub fusion regardless of the identity of a junctional residue X (the sole exception is proline at this position), the Ub fusion technique makes it possible to generate, cotranslationally, nearly any desired N-terminal residue at the N-terminus of a protein of interest. In 1986, this approach led to the discovery of the N-end rule pathway and the first degradation signals (degrons) in short-lived proteins [7], [11], [12]. The Ub fusion technique remains the method of choice for generating, *in vivo*, predetermined N-terminal residues in specific intracellular proteins. The requirement for a “technique” in this setting stems from a constraint imposed by the genetic code. Nascent proteins bear N-terminal Met (formyl-Met in bacteria). The known Met-aminopeptidases (MetAPs) cotranslationally remove N-terminal Met if a residue at position 2, to be made N-terminal by the cleavage, is Ala, Ser, Thr or another small residue [12], [13]. Larger residues at position 2, for example, bulky hydrophobic ones such as Leu or basic residues such as Arg, cannot be made N-terminal by MetAPs, a problem that can be bypassed through the Ub fusion technique.

**Figure 1:**
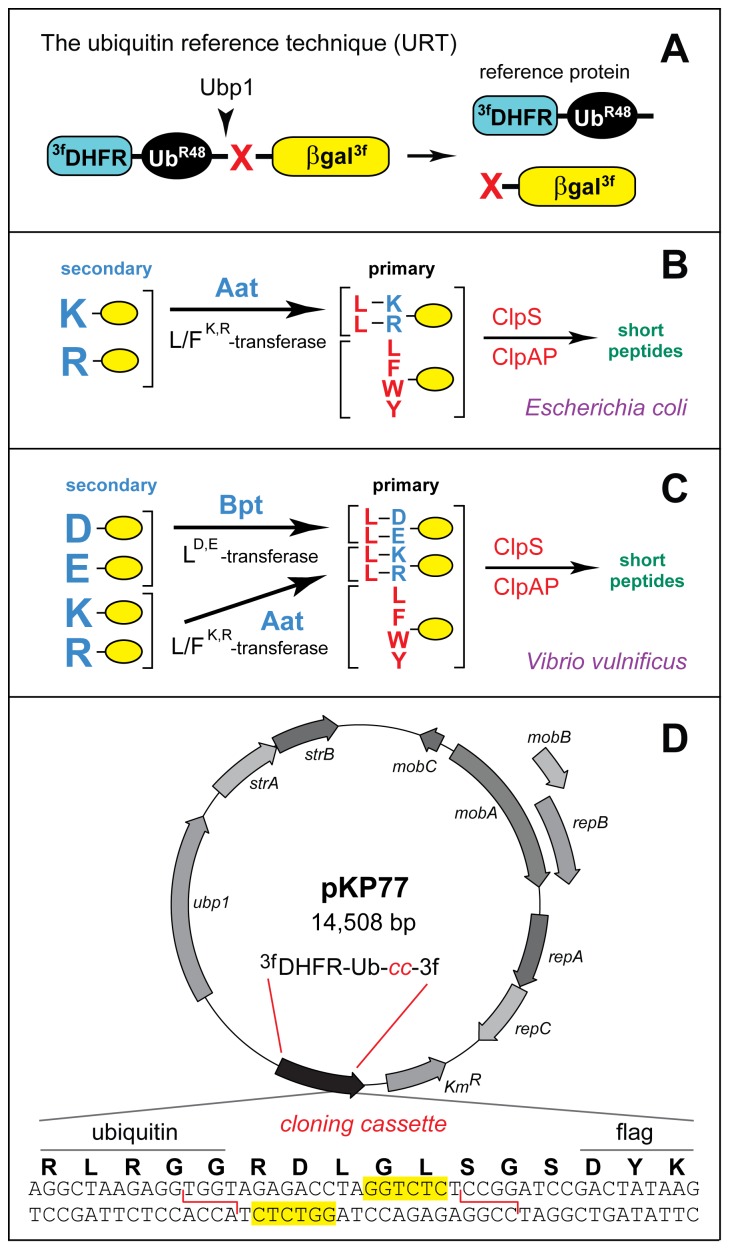
Ubiquitin reference technique (URT) and bacterial N-end rule pathways. ***A***. The Ub reference technique (URT), derived from the Ub fusion technique [9], [18], [20]. Cotranslational cleavage of a URT-based fusion by deubiquitylases (DUBs) produces, at the initially equimolar ratio, a test protein (in the present work, the C-terminally triple flag-tagged and otherwise modified *E*. *coli* X-β-galactosidase (X-βgal)) with a desired N-terminal residue and a “reference” protein such as ^3f^DHFR-Ub^R48^, a triple flag-tagged derivative of the mouse dihydrofolate reductase. In URT-based pulse-chase assays, the pulse-labeled test protein is quantified by measuring its levels relative to the levels of a stable reference at the same time point. In addition to being more accurate than pulse-chases without a reference, URT assays make it possible to detect and measure the degradation of a test protein during the pulse, i.e., before the chase [9], [20]. ***B***. The *E. coli* N-end rule pathway [12]. N-terminal residues are indicated by single-letter abbreviations for amino acids. Yellow ovals denote the rest of a protein substrate. “Primary” and “secondary” denote mechanistically distinct subsets of destabilizing N-terminal residues. The Aat L/F^R,K^-transferase conjugates largely Leu (or, to a minor extent, Phe) to N-terminal Arg or Lys. N-end rule substrates bearing the indicated primary (bulky hydrophobic) destabilizing N-terminal residues are recognized by the ClpS N-recognin and are delivered for their processive degradation to the ClpAP protease [12], [22], [23], [28]–[31]. ***C***. The N-end rule pathway in another Gram-negative bacterium, *V. vulnificus*, which contains both the Aat L/F^R,K^-transferase and the Bpt L^D,E^-transferase. As a result, N-terminal Asp and Glu, which are stabilizing (nondestabilizing) residues in *E. coli*, are secondary destabilizing residues in the *V. vulnificus* N-end rule pathway [12], [30]. ***D***. The broad host-range URT vector pKP77. It encodes the *S. cerevisiae* Ubp1 deubiquitylase (DUB) as well the ^3f^DHFR-Ub^R48^ reference protein, followed by a DNA sequence containing a cloning cassette (*cc*) as well as a sequence encoding the triple flag tag. The cloning cassette contains two inverted BsaI sites (yellow rectangles). Digestion of pKP77 with BsaI generates unique cohesive ends (indicated by red lines) that allow precise, unidirectional insertion of a sequence of interest while preventing self-ligation of the cut plasmid (see Materials and Methods). Other notations on the map denote specific bacterial genes of the parental pJRD215 plasmid (GenBank accession number JX181778).

Since 1986, several otherwise unrelated methods were invented that had in common the use of Ub fusions as a component of design [6], [9], [14]–[17]. One of them was the Ub Reference Technique (URT), which addressed the problem of data scatter in conventional pulse-chase degradation assays [14]. A URT assay is based on a fusion in which the Ub moiety is located between a downstream polypeptide (test protein) and an upstream polypeptide (a long-lived reference protein). The cotranslational cleavage of a URT fusion by DUBs after the last residue of Ub produces, at the initially equimolar ratio, a test protein with a desired N-terminal residue and a reference protein that contains the C-terminal Ub moiety. A reference protein can be, for example, ^3f^DHFR-Ub^R48^, a triple flag-tagged derivative of the mouse dihydrofolate reductase (Figure 1A). If both the reference protein and the test protein are immunoprecipitated and analyzed in a pulse-chase assay, the relative levels of the test protein can be calibrated against the long-lived reference protein in the same sample [6], [18], [19]. As a result, the URT assay can compensate for the scatter of labeling efficiency, immunoprecipitation yields, sample volumes and other sources of sample-to-sample variation. In addition to being more accurate than pulse-chases without a reference, URT makes it possible to detect and measure the degradation of a test protein during the pulse (before the chase). The latter capability of URT allows one to detect and measure the degradation of a protein that occurs either cotranslationally, i.e., before the completion of the protein's polypeptide chain, or shortly after its completion. With some proteins (including test proteins of the present work; see below), the rates of their “early” *in vivo* degradation can be much higher than the rates of their subsequent degradation, in part because of protein folding and association with other proteins.

One application of URT are measurements of protein degradation by the N-end rule pathway [6], [18], [20], [21]. This multifunctional proteolytic system recognizes proteins containing N-terminal degradation signals called N-degrons, polyubiquitylates these proteins and thereby causes their degradation by the proteasome [11], [12], [20]–[27]. The main determinant of an N-degron is a destabilizing N-terminal residue of a protein. Recognition components of the N-end rule pathway are called N-recognins. In eukaryotes, N-recognins are E3 Ub ligases that can target N-degrons. Prokaryotes (bacteria and archaea) lack the bona fide Ub system. Nevertheless, all examined bacteria, including Gram-negative *Escherichia coli* and *Vibrio vulnificus*, were found to contain the N-end rule pathway, Ub-independent versions of it. Bacterial N-end rule substrates are recognized, in particular, by the ClpS N-recognin, a small protein that appears to share ancestry with much larger eukaryotic N-recognins that act as Ub ligases. ClpS binds to bulky hydrophobic N-terminal residues and delivers targeted N-end rule substrates to the proteasome-like protease ClpAP (Figure 1B, C) [12], [22], [23], [28]–[31].

Because a Ub fusion is not cleaved at the Ub moiety in a prokaryotic cell, previous uses of the Ub fusion technique in bacteria involved an ectopic expression of a eukaryotic DUB such as the *Saccharomyces cerevisiae* Ubp1 [28]–[30]. However, neither URT nor single-plasmid designs for performing URT assays have been extended to prokaryotes so far. Here, we describe URT-based assays of the N-end rule pathway with *E. coli* and *V. vulnificus* that employ a convenient single-plasmid design, thereby extending the advantages of URT to studies of protein degradation in prokaryotes.

## Materials and Methods

### Miscellaneous Reagents

Anti-FLAG M2 Magnetic Beads (M8823) were from Sigma (St. Louis, MO, USA). Complete EDTA-free Protease Inhibitor Cocktail Tablets were from Roche (San Francisco, CA, USA). Express [^35^S] Protein Labeling Mix (1.175 Ci/mmol) was from Perkin-Elmer (Waltham, MA, USA). Methionine/Cysteine-free Synthetic Complete (″Hopkins″) Supplement Mixture (SC) was from Sunrise Science Products (San Diego, CA, USA). Difco TCBS agar was from Becton-Dickinson (Franklin Lakes, NJ, USA).

### Construction of pKP55-X and pKP77 Plasmids

*E*. *coli* DH5α (Invitrogen, Carlsbad, CA, USA)) and *E*. *coli* KPS18 [30] (Table S1) were used for cloning and maintaining plasmids. Phusion High-Fidelity DNA polymerase (New England Biolabs, Ipswich, MA, USA) was used for polymerase chain reaction (PCR). Constructs were generated using standard techniques and verified by DNA sequencing. The *S. cerevisiae* P*_GAL1_* promoter (fragment 1) was amplified by PCR using pUB23 (Table S1) [7] as a template and primers 159–162 (Table S2). DNA fragment encoding ^3f^DHFR-Ub^R48^ (fragment 2) was assembled using pcDNA3-fDHFR-UbR48-M-cMos (Table S1) [19] as a template and primers 163–170, 172 (Table S2). DNA fragment encoding a modified *E*. *coli* X-β-galactosidase (X-βgal) (fragment 3) was amplified by PCR using pUB23 as a template and primers 171, 173–175. DNA fragment encoding the C-terminal triple flag fragment (fragment 4) was assembled by annealing/elongation of primers 176–180 (Table S2). DNA fragment for producing ^3f^DHFR-Ub^R48^-*cc (cloning cassette)*-βgal^3f^ (Figure 1A) was assembled by PCR using primers 159, 180 and PCR-generated fragments 1–4 as a template. DNA sequence of the *cloning cassette* (*cc*) contained two inverted Eco3lI cleavage sites, as depicted in Figure 1D for the subsequently constructed pKP77 plasmid. The resulting fragment was cut with XmaI/BglII and subcloned into XmaI/BamHI-cut pJT70 (Table S1) [28], yielding pEco31I. The latter plasmid was cut with XbaI/PstI and the fragment encoding both Ubp1 and the URT fusion was subcloned into NsiI/NheI-cut pJRD215 (Table S1) [32], yielding pKP54. Primers 187, 188 (Table S2) were annealed in ligation buffer [33] and cloned into Eco31I-cut pKP54. The resulting pKP55-X family of plasmids, encoding both *S. cerevisiae* Ubp1 and a set of ^3f^DHFR-Ub^R48^-X-βgal^3f^ fusions (with varying identities of the junctional residue X) was transformed into *E. coli* KPS18 (*Δ(lac)_X74_ aat::minitet ΔclpA*) and plated on LB agar supplemented with kanamycin (50 µg/ml) and XGal (40 µg/ml). Blue colonies were isolated and sequenced to determine the identity of the junctional amino acid residue X in each member of the pKP55-X family (Table S3).

To construct pKP77, the DNA fragment of pKP54 encoding ^3f^DHFR-Ub^R48^ and followed by a DNA segment containing two inverted Eco31I sites was amplified by PCR using primers 160 and 208 (Table S2). The resulting 1.3 kb fragment was cut with NsiI/BspEI and subcloned into NsiI/BspEI-cut pKP54, yielding pKP77 (Figure 1D).

### Bacterial Strains and Transfer of Plasmids by Conjugation

*E*. *coli* was grown at 37°C on Luria-Bertani (LB) medium. For propagating the *dap* ^-^ strain BW29427, LB was supplemented with diaminopimelic acid (Sigma) to the final concentration of 100 µg/ml. *V*. *vulnificus* was grown at 37°C on LB or TCBS Agar (Becton-Dickinson). When used for selection, antibiotics were added to the following final concentrations: kanamycin (Km): 50 µg/ml; ampicillin (Amp) 100 µg/ml. *E*. *coli* KPS18, a null *clpA* mutant, was constructed using a previously described gene disruption strategy [30]. The conjugation-mediated transfer of mobilizable pKP55-X plasmids from *E*. *coli* into *V*. *vulnificus* was carried out as follows. *E*. *coli* donor cells (BW29427) containing a desired plasmid were grown overnight at 37°C in LB medium supplemented with diaminopimelic acid (100 µg/ml) and kanamycin (50 µg/ml). 0.1 ml of *E*. *coli* suspension was added to equal volume of an overnight culture of *V*. *vulnificus*, cells were harvested by centrifugation, washed twice with LB to remove residual antibiotics, spread on LB agar plates without selection, and incubated for 12 h at 30°C. Cells were resuspended in 1 ml of LB and aliquots of serial 10-fold dilutions were plated on TCBS agar supplemented with kanamycin (50 µg/ml) for selection of exconjugants. The resulting colonies of *V. vulnificus* were grown in LB under selective conditions and analyzed for the presence of desired plasmids either by PCR or by isolating and characterizing plasmid DNA.

### URT Pulse-Chase Assays and Immunoprecipitation

*E*. *coli* and *V*. *vulnificus* cells containing URT-based reporter plasmids were grown in LB supplemented with Km (50 µg/ml) at 37°C overnight. Cultures were diluted 1∶200 in fresh LB and grown until A_600_ of ∼0.7. 15 ml of the resulting culture were centrifuged at 5000*g* for 5 min at room temperature, washed three times with 1 ml samples of pre-warmed Pulse Medium (PM: M9 medium, pH 7.0, 0.5% glycerol, 0.5% glucose, 0.1 mM CaCl_2_, 2 mM MgSO_4_ and Methionine/Cysteine-free Synthetic Complete (SC) Mixture (Sunrise Science Products)), and resuspended in 135 µl of PM, followed by incubation at 37°C for 10 min. Proteins were then pulse-labeled with 15 µl of Express [^35^S] Protein Labeling Mix (1.175 Ci/mmol, Perkin Elmer) for 3 min at 37°C. The labeling was quenched by the addition of 0.5 ml of Chase-Medium (CM: PM supplemented with Met and Cys at 0.5 mg/ml each). The chase was carried out at 37°C. Samples (0.1 ml) were withdrawn at indicated times of chase and mixed with an equal volume of TDS buffer (1% SDS, 5 mM dithiothreitol (DTT), 50 mM Tris-HCl, pH 7.4, containing “complete protease-inhibitor mixture” (Roche)), followed by immediate freezing in liquid nitrogen. Frozen samples were directly heated at 95°C for 10 min, diluted with 10 volumes of TNN buffer (0.5% NP40, 0.25 M NaCl, 5 mM EDTA, 50 mM Tris-HCl, pH 7.4, containing “complete protease-inhibitor mixture” (Roche)) and thereafter added to 10 µl of magnetic beads linked to anti-flag antibody M2 (Sigma). The samples were incubated with rocking at 4°C for 3 hrs, followed by four washes in TNN buffer, resuspension in 20 µl of SDS-sample buffer, and incubation at 95°C for 5 min. The resulting samples were fractionated by SDS-PAGE using NuPAGE 4–12% Bis-Tris gradient gels (Invitrogen), followed by autoradiography. Quantification of autoradiograms was performed using PhosphorImager (Molecular Dynamics, Sunnyvale, CA).

## Results and Discussion

### Bacterial URT Plasmids and Protein Fusions

**Figure 2:**
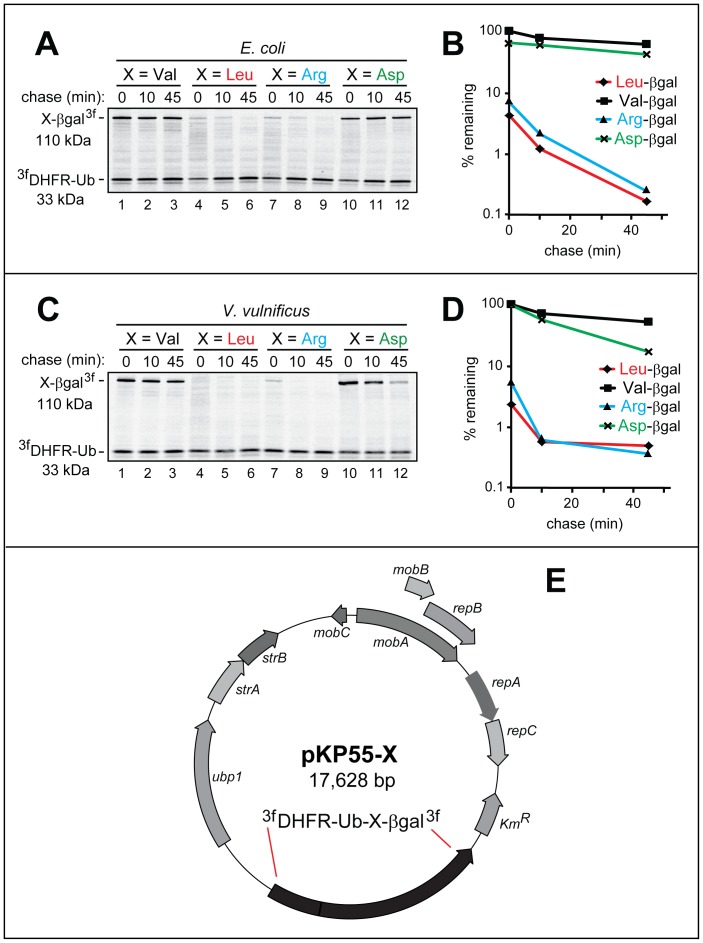
URT pulse-chase assays with model N-end rule substrates in *E. coli* and *V. vulnificus*. The set of URT-based ^3f^DHFR-Ub^R48^-X-βgal^3f^ fusions (X = Val, Leu, Arg, Asp) was assayed for the *in vivo* degradation of the released (by the yeast Ubp1 DUB) X-βgal proteins in *E. coli* (***A, B***) and in *V. vulnificus* (***C, D***) using ^35^S-pulse-chases (***A, C***) and their quantification (***B, D***), as described in Materials and Methods. The bands of the 110 kDa X-βgal test proteins and the 33 kDa ^3f^DHFR-Ub^R48^ reference protein are indicated on the left. Designations in ***B*** and ***D***: squares, Val-βgal; rhombs, Leu-βgal; triangles, Arg-βgal; crosses, Asp-βgal. ***E***. pKP55-X, encoding the *S. cerevisiae* Ubp1 DUB and ^3f^DHFR-Ub^R48^-X-βgal^3f^ URT-based fusions. Other notations on the map denote specific bacterial genes. The nucleotide sequences of pKP77 and pKP55-X are available in GenBank (JX181779 and JX181780). In addition, Table S3 contains the nucleotide sequence of pKP55-X.

Figure 1A shows a diagram of the URT-based fusion protein reporter used in the present work. The cotranslational *in vivo* cleavage of this protein by DUBs produces, at initially equimolar amounts, the C-terminally triple flag-tagged test protein X-β-galactosidase^3f^ (X-βgal^3f^) with a desired (varying) N-terminal residue X, as well as the long-lived, triple flag-tagged reference protein ^3f^DHFR-Ub^R48^ (see Introduction and Figure 1A). To extend the use of URT from eukaryotes to prokaryotes and to validate this method in bacteria using model N-end rule substrates, we constructed pKP55-X plasmids that expressed both the *S. cerevisiae* Ubp1 DUB [29] and one of the otherwise identical URT fusions ^3f^DHFR-Ub^R48^-X-βgal^3f^ that differed by the identity of their junctional residue X (Figure 2E and Table S3). Although the *E. coli* ElaD protease exhibits DUB activity *in vitro* [34], its *in vivo* DUB activity vis-á-vis URT fusions was negligible. When expressed in *E. coli*, the yeast Ubp1 DUB efficiently cleaved Ub-X-βgal fusions both in *E*. *coli* extract and *in vivo* [28], [29]. The use of one plasmid (rather than two) to express both a DUB enzyme and a URT fusion simplifies the final setup and bypasses complications of plasmid incompatibility.

The pKP55-X plasmids were derived from the broad-range, low copy plasmid pJRD215 (*E. coli* compatibility group Q (IncQ)). pJRD215 was derived, in turn, from the plasmid RSF1010 [32]. Plasmids of the IncQ group have been shown to replicate in a wide variety of Gram-negative bacteria, including *Agrobacterium*, *Alcaligenes*, *Bacillus, Pseudomonas*, *Rhizobium, Rhodobacter*, *Staphylococcus*, and *Vibrio* [35], [36]. These plasmids can be efficiently transferred between species through conjugation in the presence of a conjugation-proficient plasmid such as RP4 [37]. In addition, IncQ plasmids are compatible with other broad-range replicons of the IncP, IncW, and pBHR-pBBR groups [38].

A URT-based protein fusion encoded by a pKP55-X plasmid comprised the following elements, starting from the N-terminus: (i) The triple-flag tag (DYKDDDDKG)_3_, with the Ser-Gly-Ser (SGS) linker sequences flanking the middle flag repeat, and with the first flag repeat preceded by N-terminal Met. (ii) The 21 kDa mouse DHFR moiety. (iii) Ub^R48^, a modified Ub moiety in which Lys48 of Ub had been replaced by Arg, thereby precluding ubiquitylation of the Ub moiety at position 48. This modification of Ub was irrelevant to its use in Ub-lacking prokaryotic settings but may be helpful for applications of these URT fusions in eukaryotes, by decreasing the probability of the ^3f^DHFR-Ub^R48^ moiety acting as a substrate of Ub-conjugating enzymes. (iv) A varying (through site-directed mutagenesis) residue X after the last residue of Ub^R48^. (v) The 116-kDa modified *E. coli* βgal moiety lacking the first 6 residues of wild-type βgal and bearing a 45-residue N-terminal sequence derived from *E. coli* Lac repressor [7]–[9], [18] (see Materials and Methods). (vi) C-terminal triple flag tag (Figures 1A and 2E). The yeast Ubp1 DUB was expressed from the native *S. cerevisiae* P*_UBP1_* promoter, which is active in examined bacteria [29]. URT-based ^3f^DHFR-Ub^R48^-X-βgal^3f^ fusions were expressed from the *S. cerevisiae* P*_GAL10_* promoter, which acts as a weak constitutive promoter in examined bacteria [29], [30].

### URT-Based N-End rule Reporters in *E*. *coli* and *V*. *vulnificus*

*Vibrio vulnificus* is a Gram-negative human pathogen naturally found in marine environments, including shellfish [30]. We used the URT reporter (Figure 1A) to examine the previously characterized N-end rule pathway in *E. coli* and *V. vulnificus*. In the first set of URT assays, the pKP55-X plasmids were transformed into *E*. *coli*. Cells expressing both yeast Ubp1 and ^3f^DHFR-Ub^R48^-X-βgal^3f^ (X = Val, Leu, Arg, Asp) were labeled with ^35^S-Met/Cys for 3 min at 37°C, followed by chase, immunoprecipitation with anti-flag antibody, SDS-PAGE, autoradiography and quantification (Figure 2A, B, E).

The logic of these assays involves a comparison between the degradation rates of a protein bearing a destabilizing N-terminal residue and an otherwise identical protein with an N-terminal residue such as Val, which is not recognized by the N-end rule pathway [12]. Both Leu-βgal, whose N-terminal Leu is a primary destabilizing residue (see the legend to Figure 1 for N-end rule terminology) and Arg-βgal, whose N-terminal Arg is a secondary destabilizing residue in bacteria, were approximately equally short-lived in *E*. *coli*, with the posttranslational t_1/2_ of ∼3 min (Figure 2A, B). Strikingly, more than 90% of pulse-labeled Leu-βgal and Arg-βgal were degraded during the 3-min pulse (i.e., before the chase), in comparison to the otherwise identical Val-βgal and Asp-βgal, which were stable under the same conditions (Figure 2A, B). Unmodified N-terminal Val is not recognized as a destabilizing residue by the N-end rule pathway [12]. In contrast, N-terminal Asp is a stabilizing residue in *E. coli* but a secondary destabilizing residue in *V. vulnificus*, as described below. A stabilizing N-terminal residue is defined as a residue that is not recognized, in a stated *in vivo* setting, as a destabilizing residue.

BW29427 *E*. *coli* was used as a donor strain to transfer pKP55-X plasmids from *E. coli* to *V*. *vulnificus*. Exconjugants bearing desired pKP55-X plasmids (X = Val, Leu, Arg, Asp) were selected as described in Materials and Methods. URT-based ^35^S-pulse-chase assays with *V*. *vulnificus* were carried out and quantified as described above for *E*. *coli* (Figure 2C, D). Both Leu-βgal and Arg-βgal were rapidly degraded by the *V*. *vulnificus* N-end rule pathway, in contrast to Val-βgal (Figure 2C, D). Asp-βgal was long-lived in *E*. *coli* (Figure 2A, B) but relatively short-lived in *V*. *vulnificus*, owing to the presence, in the latter bacterium, of the previously identified and characterized Leu-tRNA-protein transferase (L^D,E^-transferase) (Figure 2C, D. This enzyme, present in some bacteria and absent from examined eukaryotes, is encoded by the *bpt* gene and utilizes Leu-tRNA to conjugate Leu to N-terminal Asp, Glu and (possibly) oxidized Cys, thereby making these residues destabilizing in the *V. vulnificus* N-end rule pathway (Figure 1, B, C) [30].

### URT Vector

To facilitate the use of URT assays in bacteria, we also constructed pKP77, a generally applicable URT expression vector (Figure 1D). Through the use of this plasmid, any test protein or its fragment can be expressed in a bacterium as a part of a ^3f^DHFR-Ub^R48^-X-protein^3f^ fusion and examined in URT-based pulse-chase assays. The pKP77 plasmid contains DNA segments encoding yeast Ubp1 as well as the ^3f^DHFR-Ub^R48^ part of a ^3f^DHFR-Ub^R48^-X-protein^3f^ fusion, with “protein” being any desired polypeptide, and with X being a varying residue. Given strict constraints on distances between Ub-proximal elements of a final multipartite fusion, subcloning into a URT vector can be technically cumbersome. To facilitate the cloning, pKP77 was designed to contain two (appropriately arranged) inverted BsaI sites [GGTCTC(1/5)] (Figure 1D) [39]. Digestion of pKP77 with BsaI generates unique cohesive ends that allow a precise, unidirectional insertion of a DNA sequence of interest while preventing self-ligation of the vector. If a DNA sequence to be inserted has internal BsaI sites, other endonucleases of the same kind can be used to generate required cohesive ends, for example, BbsI [GAAGAC (2/6)], BsmBI [CGTCTC (1/5)], or BspMI [ACCTGC (4/8)] [39].

pKP77 and pKPP55-X are diagrammed in Figures 1D and 2E, respectively. The corresponding nucleotide sequences are available under the GenBank accession numbers JX181779 and JX181780. The entire sequence of pKPP55-X is shown in Table S3. Specific URT designs of the present work and their demonstrated utility (Figures 1 and 2) should facilitate the extension of URT-based technologies to studies of protein degradation in prokaryotes.

## Supporting Information

Table S1 **Bacterial strains and plasmids used in this study.**
(DOCX)

Table S2 **Oligonucleotide primers used in this study.**
(DOCX)

Table S3 **The nucleotide sequence of the pKP55-X plasmid.** The notation “xxx” denotes a varying codon encoding an amino acid residue X at the Ub-protein junction (see the main text).
(DOCX)
