# Supplementary material for: Ubiquitin Reference Technique and Its Use in Ubiquitin-Lacking Prokaryotes
Source: PLoS One. 2013 Jun 25;8(6):e67952. doi: 10.1371/journal.pone.0067952 (PMC3692480; doi:10.1371/journal.pone.0067952)
Supplement: Table S1 — Bacterial strains and plasmids used in this study. (DOCX) [file pone.0067952.s001.docx]

**Table S1.** Bacterial strains and plasmids used in this study.

|  | **Description** | **Source or Reference** |
| --- | --- | --- |
| ***E. coli Strains*** |  |  |
| KPS18 | *hsdR2 hsdM^+^ hsdS^+^ araD139 Δ(ar-leu)7697 Δ(lac)_X74_ galE15 galK16 rpsL mcrA mcrB1 aat::minitet ΔclpA* | [[1](#_ENREF_1)] |
| BW29427 | thrB1004 pro thi rpsL(Str^R^) hsdS lacZΔM15 RP4-1360 Δ(araBAD)567 ΔdapA1341::[erm pir (wt)] | A gift from B.L. Wanner (Purdue University, Lafayette, IN, USA) |
| DH5α | *φ80 lacZΔM15 endA1 recA1 gyrA96 thi-1 hsdR17 (r_k_^-^ , m_k_^-^ ) relA1 supE44 deoR ∆(lacZYA–argF)U169* | Promega |
|  |  |  |
| ***V. vulnificus* Strains** |  |  |
| C7184 | Virulent, translucent variant | [[2](#_ENREF_2)] |
|  | | |
| **Plasmids** |  |  |
| pJT70 | Amp^R^; pUC19-based plasmid with 2.8-kb BamHI/SalI insert containing *S. cerevisiae* Ubp1 | [[3](#_ENREF_3)] |
| pJRD215 | Sm^R^, Km^R^, IncQ *ori*, broad host range vector, *ori*T of RP4, *ori*V | [[4](#_ENREF_4)] |
| pUB23X | Amp^R^; plasmid encoding Ub-X-βgal under the control of the yeast GAL10 promoter (leaky expression in *E. coli*) | [[5](#_ENREF_5)] |
| pcDNA3-fDHFR-UbR48-M-cMos | Amp^R^; Neo^R^; pcDNA3.0-based plasmid encoding flag-DHFR-ha-Ub (K48R)-Met-cMos under the control of CMV promoter | [[6](#_ENREF_6)] |
| pKP55-X (a set of plasmids) | Sm^R^, Km^R^, broad host range URT reporter plasmid encoding *S. cerevisiae* Ubp1 and ^3f^DHFR-Ub-X-βgal^3f^ fusion under the control of the GAL10 promoter.  In this set of plasmids, the junctional amino acid residue X can be any residue, as described in the main text. | This study |
| pKP54 | Sm^R^, Km^R^, broad host range URT cloning vector encoding *S. cerevisiae* Ubp1 and ^3f^DHFR-Ub-sc-βgal^3f^ cassette under the control of the GAL10 promoter. This plasmid is useful for cloning different 5’ extension for βgal gene | This study |
| pKP77 | SmR, KmR, broad host range URT cloning vector encoding *S. cerevisiae* Ubp1 and 3fDHFR-Ub-sc-3f cassette under the control of the GAL10 promoter | This study |

**References**

1. Graciet E, Hu RG, Piatkov K, Rhee JH, Schwarz EM, et al. (2006) Aminoacyl-transferases and the N-end rule pathway of prokaryotic/eukaryotic specificity in a human pathogen. Proc Natl Acad Sci USA 103: 3078-3083.

2. Simpson LM, White VK, Oliver JD (1987) Correlation between virulence and colony morphology in Vibrio vulnificus. Infect Immun 55: 269-272.

3. Tobias JW, Varshavsky A (1991) Cloning and functional analysis of the ubiquitin-specific protease gene UBP1 of Saccharomyces cerevisiae. J Biol Chem 266: 12021-12028.

4. Davison J, Heusterspreute M, Chevalier N, Ha-Thi V, Brunel F (1987) Vectors with restriction site banks. V. pJRD215, a wide-host-range cosmid vector with multiple cloning sites. Gene 51: 275-280.

5. Bachmair A, Finley D, Varshavsky A (1986) *In vivo* half-life of a protein is a function of its amino-terminal residue. Science 234: 179-186.

6. Sheng J, Kumagai A, Dunphy WG, Varshavsky A (2002) Dissection of c-MOS degron. EMBO J 21: 6061-6071.
