# Supplementary material for: Ubiquitin Reference Technique and Its Use in Ubiquitin-Lacking Prokaryotes
Source: PLoS One. 2013 Jun 25;8(6):e67952. doi: 10.1371/journal.pone.0067952 (PMC3692480; doi:10.1371/journal.pone.0067952)
Supplement: Table S2 — Oligonucleotide primers used in this study. (DOCX) [file pone.0067952.s002.docx]

**Table S2.** Oligonucleotide primers used in this study.

| **Name** | Sequence, 5’ to 3’ |
| --- | --- |
| 159 | TATATAAGATCTGCGGCCGCCCGATCCAAATCATCGCTC |
| 160 | ACGACACATGATCATATAGCATGCATG |
| 161 | CATGCATGCTATATGATCATGTGTCGT |
| 162 | TCACCCATGGTATATCTCCTTCTTAAAGTAAAGTGGGAGGGAGAG |
| 163 | GAAGGAGATATACCATGGGTGACTATAAGGATGATGACGACAAAG |
| 164 | TCTTTGTAATCGGAACCAGAGCCTTTGTCGTCATCATCCTTATAG |
| 165 | GCTCTGGTTCCGATTACAAAGATGACGACGATAAGGGGAGCGGCT |
| 166 | ACTTGTCATCGTCGTCCTTGTAGTCAGAGCCGCTCCCCTTATCGT |
| 167 | GGCTCTGACTACAAGGACGACGATGACAAGTCTGGATCCCAT |
| 168 | GATGACAAGTCTGGATCCCATATGGTTCGACCATTGAACTGCATC |
| 169 | TGGCAAGAACGGTGACCTACCCTGGC |
| 170 | GCCAGGGTAGGTCACCGTTCTTGCCA |
| 171 | GCTAAGTGAGACCTAGGTCTCCTTGGCTGTTGCCCGTCTCACTGG |
| 172 | CAGCCAAGGAGACCTAGGTCTCACTTAGCACAAGATGTAAGGTGG |
| 173 | TATCGACGGTTTCCACATGGGGATTGGTG |
| 174 | CACCAATCCCCATGTGGAAACCGTCGATA |
| 175 | TCATCCTTATAGTCGGATCCGGATTTTTGACACCAGACCAACTG |
| 176 | CGGATCCGACTATAAGGATGATGACGACAAAGGCTCTGGTTCCGA |
| 177 | CCCCTTATCGTCGTCATCTTTGTAATCGGAACCAGAGCCTTTGTC |
| 178 | CAAAGATGACGACGATAAGGGGAGCGGCTCTGACTACAAGGACGA |
| 179 | GGCTCTGACTACAAGGACGACGATGACAAGTAATCTAGACCCGGG |
| 180 | CGCGTACCCGGGTCTAGATTACTTGTCATCG |
| 187 | TAAGGCTAAGAGGCGGCNNSCACGGATCCGGAGC |
| 188 | CCAAGCTCCGGATCCGTGSNNGCCGCCTCTTAGC |
| 208 | TTTTTTCCGGAGAGACCTAGGTCTCTACCACCTCTTAGCCTTAGCACAAGATGTAAGGTG |
