# Supplementary material for: Ubiquitin Reference Technique and Its Use in Ubiquitin-Lacking Prokaryotes
Source: PLoS One. 2013 Jun 25;8(6):e67952. doi: 10.1371/journal.pone.0067952 (PMC3692480; doi:10.1371/journal.pone.0067952)
Supplement: Table S3 — The nucleotide sequence of the pKP55-X plasmid. The notation “xxx” denotes a varying codon encoding an amino acid residue X at the Ub-protein junction (see the main text). (DOCX) [file pone.0067952.s003.docx]

**Table S3.** The nucleotide sequence of the pKP55-X plasmid. The notation “xxx” denotes a varying codon encoding an amino acid residue X at the Ub-protein junction (see the main text).

| GCTAGATTACTTGTCATCGTCGTCCTTGTAGTCAGAGCCGCTCCCCTTATCGTCGTCATCTTTGTAATCGGAACCAGAGCCTTTGTCGTCATCATCCTTATAGTCGGATCCGGATTTTTGACACCAGACCAACTGGTAATGGTAGCGACCGGCGCTCAGCTGGAATTCCGCCGATACTGACGGGCTCCAGGAGTCGTCGCCACCAATCCCCATGTGGAAACCGTCGATATTCAGCCATGTGCCTTCTTCCGCGTGCAGCAGATGGCGATGGCTGGTTTCCATCAGTTGCTGTTGACTGTAGCGGCTGATGTTGAACTGGAAGTCGCCGCGCCACTGGTGTGGGCCATAATTCAATTCGCGCGTCCCGCAGCGCAGACCGTTTTCGCTCGGGAAGACGTACGGGGTATACATGTCTGACAATGGCAGATCCCAGCGGTCAAAACAGGCGGCAGTAAGGCGGTCGGGATAGTTTTCTTGCGGCCCTAATCCGAGCCAGTTTACCCGCTCTGCTACCTGCGCCAGCTGGCAGTTCAGGCCAATCCGCGCCGGATGCGGTGTATCGCTCGCCACTTCAACATCAACGGTAATCGCCATTTGACCACTACCATCAATCCGGTAGGTTTTCCGGCTGATAAATAAGGTTTTCCCCTGATGCTGCCACGCGTGAGCGGTCGTAATCAGCACCGCATCAGCAAGTGTATCTGCCGTGCACTGCAACAACGCTGCTTCGGCCTGGTAATGGCCCGCCGCCTTCCAGCGTTCGACCCAGGCGTTAGGGTCAATGCGGGTCGCTTCACTTACGCCAATGTCGTTATCCAGCGGTGCACGGGTGAACTGATCGCGCAGCGGCGTCAGCAGTTGTTTTTTATCGCCAATCCACATCTGTGAAAGAAAGCCTGACTGGCGGTTAAATTGCCAACGCTTATTACCCAGCTCGATGCAAAAATCCATTTCGCTGGTGGTCAGATGCGGGATGGCGTGGGACGCGGCGGGGAGCGTCACACTGAGGTTTTCCGCCAGACGCCACTGCTGCCAGGCGCTGATGTGCCCGGCTTCTGACCATGCGGTCGCGTTCGGTTGCACTACGCGTACTGTGAGCCAGAGTTGCCCGGCGCTCTCCGGCTGCGGTAGTTCAGGCAGTTCAATCAACTGTTTACCTTGTGGAGCGACATCCAGAGGCACTTCACCGCTTGCCAGCGGCTTACCATCCAGCGCCACCATCCAGTGCAGGAGCTCGTTATCGCTATGACGGAACAGGTATTCGCTGGTCACTTCGATGGTTTGCCCGGATAAACGGAACTGGAAAAACTGCTGCTGGTGTTTTGCTTCCGTCAGCGCTGGATGCGGCGTGCGGTCGGCAAAGACCAGACCGTTCATACAGAACTGGCGATCGTTCGGCGTATCGCCAAAATCACCGCCGTAAGCCGACCACGGGTTGCCGTTTTCATCATATTTAATCAGCGACTGATCCACCCAGTCCCAGACGAAGCCGCCCTGTAAACGGGGATACTGACGAAACGCCTGCCAGTATTTAGCGAAACCGCCAAGACTGTTACCCATCGCGTGGGCGTATTCGCAAAGGATCAGCGGGCGCGTCTCTCCAGGTAGCGAAAGCCATTTTTTGATGGACCATTTCGGCACAGCCGGGAAGGGCTGGTCTTCATCCACGCGCGCGTACATCGGGCAAATAATATCGGTGGCCGTGGTGTCGGCTCCGCCGCCTTCATACTGCACCGGGCGGGAAGGATCGACAGATTTGATCCAGCGATACAGCGCGTCGTGATTAGCGCCGTGGCCTGATTCATTCCCCAGCGACCAGATGATCACACTCGGGTGATTACGATCGCGCTGCACCATTCGCGTTACGCGTTCGCTCATCGCCGGTAGCCAGCGCGGATCATCGGTCAGACGATTCATTGGCACCATGCCGTGGGTTTCAATATTGGCTTCATCCACCACATACAGGCCGTAGCGGTCGCACAGCGTGTACCACAGCGGATGGTTCGGATAATGCGAACAGCGCACGGCGTTAAAGTTGTTCTGCTTCATCAGCAGGATATCCTGCACCATCGTCTGCTCATCCATGACCTGACCATGCAGAGGATGATGCTCGTGACGGTTAACGCCTCGAATCAGCAACGGCTTGCCGTTCAGCAGCAGCAGACCATTTTCAATCCGCACCTCGCGGAAACCGACATCGCAGGCTTCTGCTTCAATCAGCGTGCCGTCGGCGGTGTGCAGTTCAACCACCGCACGATAGAGATTCGGGATTTCGGCGCTCCACAGTTTCGGGTTTTCGACGTTCAGACGTAGTGTGACGCGATCGGCATAACCACCACGCTCATCGATAATTTCACCGCCGAAAGGCGCGGTGCCGCTGGCGACCTGCGTTTCACCCTGCCATAAAGAAACTGTTACCCGTAGGTAGTCACGCAACTCGCCGCACATCTGAACTTCAGCCTCCAGTACAGCGCGGCTGAAATCATCATTAAAGCGAGTGGCAACATGGAAATCGCTGATTTGTGTAGTCGGTTTATGCAGCAACGAGACGTCACGGAAAATGCCGCTCATCCGCCACATATCCTGATCTTCCAGATAACTGCCGTCACTCCAGCGCAGCACCATCACCGCGAGGCGGTTTTCTCCGGCGCGTAAAAATGCGCTCAGGTCAAATTCAGACGGCAAACGACTGTCCTGGCCGTAACCGACCCAGCGCCCGTTGCACCACAGATGAAACGCCGAGTTAACGCCATCAAAAATAATTCGCGTCTGGCCTTCCTGTAGCCAGCTTTCATCAACATTAAATGTGAGCGAGTAACAACCCGTCGGATTCTCCGTGGGAACAAACGGCGGATTGACCGTAATGGGATAGGTCACGTTGGTGTAGATGGGCGCATCGTAACCGTGCATCTGCCAGTTTGAGGGGACGACGACAGTATCGGCCTCAGGAAGATCGCACTCCAGCCAGCTTTCCGGCACCGCTTCTGGTGCCGGAAACCAGGCAAAGCGCCATTCGCCATTCAGGCTGCGCAACTGTTGGGAAGGGCGATCGGTGCGGGCCTCTTCGCTATTACGCCAGCTGGCGAAAGGGGGATGTGCTGCAAGGCGATTAAGTCGGGAAACCTGTCGTGCCAGCTGCATTAATGAATCGGCCAACGCGCGGGGAGAGGCGGTTTGCGTATTGGGCGCCAGGGTGGTTTTTCTTTTCACCAGTGAGACGGGCAACAGCCAAGCTCCGGATCCGTG**xxx**GCCGCCTCTTAGCCTTAGCACAAGATGTAAGGTGGACTCCTTCTGAATGTTGTAATCAGACAGCGTTCTACCGTCTTCTAGCTGCCTACCGGCAAAGATCAATCTTTGTTGATCTGGAGGGATACCTTCCTTGTCTTGAATTTTCGACTTAACGTTGTCGATGGTATCGGAAGATTCAACTTCCAATGTTATGGTTTTACCGGTCAAAGTCTTGACGAAAATCTGCATGGTCGACCCACTACCCGCAAGGAAAGCGGCGTAATCTGGGACATCGTATGGGTAGGTACCGTCTTTCTTCTCGTAGACTTCAAACTTATACTTGATGCCTTTTTCCTCCTGGACCTCAGAGAGGACGCCTGGGTATTCTGGGAGAAGTTTATATTTCCCCAAATCAATTTCTGGGAAAAACGTGTCACTTTCAAATTCCTGCATGATCCTTGTCACAAAGAGTCTAAGGTGGCCTGGTTGATTCATGGCTTCCTGGTAAACAGAACTGCCTCCGACTATCCAAACCATGTCTACTTTACTTGCCAATTCCGGTTGTTCAATAAGTCTTAAGGCATCATCCAAACTTTTGGCAAGAAAATGAGCTCCTCGTGGTGGTTCTTTGAGTTCTCTACTGAGAACTATATTAATTCTGTCCTTTAAAGGTCGATTCTTCTCAGGAATGGAGAACCAGGTTTTCCTACCCATAATCACCAGATTCTGTTTACCTTCCACTGAAGAGGTTGTGGTCATTCTTTGGAAGTACTTGAACTCGTTCCTGAGCGGAGGCCAGGGTAGGTCACCGTTCTTGCCAATCCCCATATTTTGGGACACGGCGACGATGCAGTTCAATGGTCGAACCATATGGGATCCAGACTTGTCATCGTCGTCCTTGTAGTCAGAGCCGCTCCCCTTATCGTCGTCATCTTTGTAATCGGAACCAGAGCCTTTGTCGTCATCATCCTTATAGTCACCCATGGTATATCTCCTTCTTAAAGTAAAGTGGGAGGGAGAGTTCGAGAGTTCTTTTCGTATTGTAGTAGATAGAAGATATACTTATCCTTATCCGTACAGTTCTTGCTCTTTCTGAAGTAGCACTTTCGGAAGAAGAAAGATAAGCAGGCGAATTGATCCGGTAATTTAGTGTGTGTATTTGTGTTTGCGTGTCTATAGAAGTATAGTAATTTATGCTACAAAGGACCTAATGTATAAGGAAAGAATATTTAGAGAAAAGAAGAAAACAAGAGTTTTATATACATACAGAGCACATGCATGCTATATGATCATGTGTCGTCGCACACATATATATATGCCTGTATGTGTCAGCACTAAAGTTGCCTGGCCATCCACGCTATATACACGCCTGGCGGATCTGCTCGAGGTCGATTAGCCTAAAAAAACCTTCTCTTTGGAACTTTCAGTAATACGCTTAACTGCTCATTGCTATATTGAAGTACGGATTAGAAGCCGCCGAGCGGGTGACAGCCCTCCGAAGGAAGACTCTCCTCCGTGCGTCCTCGTCTTCACCGGTCGCGTTCCTGAAACGCAGATGTGCCTCGCGCCGCACTGCTCCGAACAATAAAGATTCTACAATACTAGCTTTTATGGTTATGAAGAGGAAAATTGGCAGTAACCTGGCCCCACAACCTTCAATGAACGAATCAATTACCACCATAGGATGATAATGCGATTAGTTTTTTAGCCTATTTCTGGGGTATTATCAGCGAGCGATGATTTGGATCGGGCGGCCGCAGATCTGTAAGCCGTAATGTTTTTCCCATAAACGTCACTGAATGTCCATATATCATACTACCAGTAAGCGCCTGTTGGAAACCTTCCTACCTTCATACGTGTATTCTGCTATATCCCTTCTTTTCCTCTTTACGTACAGAATGACATCTATGTAGGCAAGTTTCTTTTTCCATCATTGGAAAACGAAAATACATTTTTCACTAGCACCACAAAATTTTCAAGATGGCCAGGCTAGTTAAATCTATTATACAATAATTATTGGTTATATATTTAAAAAACAGAAACATTTTTTGCTTTAAAAGCACGCCATAGTATTGCTTTTAATATTGTGATCTGCGTCCTTTTTTTTCTCAGGAAAAAAAAATTTTATACACATTCAAAGAATAGAAGCGATTGTCAAAATTCGCTTCTCCTTTCTTTTCCATTATAACGTCTGATCATTTTACGTCTTCAGTGCCCTCCCTTGTTCGAAACTAGATACTTTCGAACACTTCTCCCCTTTTAATCTACAAAATTTTGTATGGATTTGTTTATTGAAAGCAAGATAAACAGTTTATTACAATTTTTATTTGGTTCCCGACAGGATTTTTTGAGAAATTTTAAAACTTGGAGTAACAACAATAACAATCTATCGATTTATTTATTAATTTTTGGCATAGTAGTATTTTTTTATAAAAAACCAGACCATCTAAACTACATTGTTGAGAGCGTTAGTGAAATGACAACAAACTTCAGAAATAATAATAGCCTTAGCCGTTGGTTGCCCAGAAGTAAGTTTACCCACTTAGACGAAGAGATCTTGAAAAGAGGTGGTTTCATTGCTGGTTTAGTTAATGATGGTAACACTTGTTTTATGAACTCTGTTTTGCAATCATTGGCATCATCCAGAGAATTAATGGAGTTCTTGGACAATAATGTCATAAGGACCTATGAGGAGATAGAACAAAATGAACACAATGAAGAAGGAAACGGGCAAGAATCTGCTCAAGATGAAGCCACTCATAAGAAAAACACTCGTAAGGGTGGCAAAGTTTATGGTAAGCATAAGAAGAAATTGAATAGGAAGTCAAGTTCGAAAGAAGACGAAGAAAAGAGCCAGGAGCCAGATATCACTTTCAGTGTCGCCTTAAGGGATCTACTTTCTGCCTTAAATGCGAAGTATTATCGGGATAAACCCTATTTCAAAACCAATAGTTTATTGAAAGCAATGTCCAAATCTCCAAGAAAAAATATTCTTCTTGGCTACGACCAAGAGGACGCGCAAGAATTCTTCCAGAACATACTAGCCGAGTTGGAAAGTAACGTTAAATCATTGAATACTGAAAAACTAGATACCACTCCAGTTGCGAAATCAGAATTACCCGATGATGCTTTAGTAGGTCAACTTAACCTTGGTGAAGTTGGCACTGTTTACATTCCAACTGAACAGATTGATCCTAACTCTATACTACATGACAAGTCCATTCAAAATTTCACACCTTTCAAACTAATGACTCCTTTAGATGGTATCACGGCAGAAAGAATTGGTTGTTTACAGTGTGGTGAGAACGGTGGCATAAGATATTCCGTATTTTCGGGATTAAGCTTAAATTTACCGAACGAGAATATTGGTTCCACTTTAAAATTATCTCAGTTATTAAGCGACTGGAGTAAACCTGAAATCATCGAAGGCGTAGAATGTAACCGTTGTGCCCTCACAGCAGCGCACTCTCATTTATTTGGTCAGTTGAAAGAATTTGAAAAAAAACCTGAGGGTTCGATCCCAGAAAAGTTAATTAACGCTGTAAAAGATAGGGTCCATCAAATCGAAGAAGTTCTTGCCAAACCAGTTATTGACGATGAAGATTATAAGAAGTTGCATACAGCAAATATGGTACGTAAATGCTCTAAATCTAAGCAGATTTTAATATCAAGACCTCCACCATTATTATCCATTCATATCAACAGATCCGTATTTGATCCAAGAACGTACATGATTAGAAAAAATAACTCGAAAGTATTGTTTAAGTCAAGGTTGAATCTTGCCCCATGGTGTTGTGATATTAATGAAATCAATTTGGATGCTCGTTTGCCAATGTCAAAAAAGGAAAAAGCTGCGCAACAAGATTCAAGTGAAGATGAAAACATTGGCGGTGAATACTATACGAAATTACATGAACGCTTCGAGCAGGAATTTGAAGACAGCGAGGAAGAAAAAGAATACGATGACGCAGAGGGGAACTATGCGTCTCATTACAATCATACCAAGGATATCAGTAACTATGATCCCCTAAACGGTGAAGTCGATGGCGTGACATCCGATGATGAAGATGAGTACATTGAAGAAACCGATGCTTTAGGGAATACAATCAAAAAAAGGATCATAGAACATTCTGATGTTGAAAACGAGAATGTAAAAGATAATGAAGAACTGCAAGAAATCGACAATGTGAGCCTTGACGAACCAAAGATCAATGTTGAAGATCAACTAGAAACATCATCTGATGAGGAAGATGTTATACCAGCTCCACCTATCAATTATGCTAGGTCATTTTCCACAGTTCCAGCCACTCCATTGACATATTCATTGCGCTCTGTCATTGTTCACTACGGTACCCATAATTATGGTCATTACATTGCATTTAGAAAATACAGGGGTTGTTGGTGGAGAATATCTGATGAGACTGTGTACGTTGTGGACGAAGCTGAAGTCCTTTCAACACCCGGTGTATTTATGTTATTTTACGAATATGACTTTGATGAAGAAACTGGGAAGATGAAGGATGATTTGGAAGCTATTCAGAGTAATAATGAAGAAGATGATGAAAAAGAGCAGGAGCAAAAAGGAGTCCAGGAGCCAAAGGAAAGCCAAGAGCAAGGAGAAGGTGAAGAGCAAGAGGAAGGTCAAGAGCAGATGAAGTTCGAGAGAACAGAAGACCATAGAGATATTTCTGGTAAAGATGTAAACTAAGTTATAAATACGATATCCGTAATTGTGTAAATAACAATAACTATAATTAAATTGAATAATTAAAAGTCTGTCGACCTGCATATGATCTTAAGGCCTAGGTCTAGAGTCTTTGTTTTGACGCCATTAGCGTACGTAACAATCCTCGTTAAAGGACAAGGACCTGAGCGGAAGTGTATCGTACAGTAGACGGAGTATACTAGTATAGTCTATAGTCCGTGGAATTATTATATTTATCTCCGACGATATTCTCATCAGTGAAATCCAGGGGAATTCTCATGTTTGACAGCTTATCATCGATAAGCTTTAATGCGGTAGTTTATCACAGTTAAATTGCTAACGCAGTCAGGCACCGTGTATGAAATCTAACAATGCGCTCATCGTCATCCTCGGCACCGTCACCCTGGATGCTGTAGGCATAGGCTTGGTTATGCCGGTACTGCCGGGCCTCTTGCGGGATAACTGCACATTCGGGATATTTCTCTATATTCGCGCTTCATCAGAAAACTGAAGGAACCTCCATTGAATCGAACTAATATTTTTTTTGGTGAATCGCATTCTGACTGGTTGCCTGTCAGAGGCGGAGAATCTGGTGATTTTGTTTTTCGACGTGGTGACGGGCATGCCTTCGCGAAAATCGCACCTGCTTCCCGCCGCGGTGAGCTCGCTGGAGAGCGTGACCGCCTCATTTGGCTCAAAGGTCGAGGTGTGGCTTGCCCCGAGGTCATCAACTGGCAGGAGGAACAGGAGGGTGCATGCTTGGTGATAACGGCAATTCCGGGAGTACCGGCGGCTGATCTGTCTGGAGCGGATTTGCTCAAAGCGTGGCCGTCAATGGGGCAGCAACTTGGCGCTGTTCACAGCCTATCGGTTGATCAATGTCCGTTTGAGCGCAGGCTGTCGCGAATGTTCGGACGCGCCGTTGATGTGGTGTCCCGCAATGCCGTCAATCCCGACTTCTTACCGGACGAGGACAAGAGTACGCCGCTGCACGATCTTTTGGCTCGTGTCGAACGAGAGCTACCGGTGCGGCTCGACCAAGAGCGCACCGATATGGTTGTTTGCCATGGTGATCCCTGCATGCCGAACTTCATGGTGGACCCTAAAACTCTTCAATGCACGGGTCTGATCGACCTTGGGCGGCTCGGAACAGCAGATCGCTATGCCGATTTGGCACTCATGATTGCTAACGCCGAAGAGAACTGGGCAGCGCCAGATGAAGCAGAGCGCGCCTTCGCTGTCCTATTCAATGTATTGGGGATCGAAGCCCCCGACCGCGAACGCCTTGCCTTCTATCTGCGATTGGACCCTCTGACTTGGGGTTGATGTTCATGCCGCCTGTTTTTCCTGCTCATTGGCACGTTTCGCAACCTGTTCTCATTGCGGACACCTTTTCCAGCCTCGTTTGGAAAGTTTCATTGCCAGACGGGACTCCTGCAATCGTCAAGGGATTGAAACCTATAGAAGACATTGCTGATGAACTGCGCGGGGCCGACTATCTGGTATGGCGCAATGGGAGGGGAGCAGTCCGGTTGCTCGGTCGTGAGAACAATCTGATGTTGCTCGAATATGCCGGGGAGCGAATGCTCTCTCACATCGTTGCCGAGCACGGCGACTACCAGGCGACCGAAATTGCAGCGGAACTAATGGCGAAGCTGTATGCCGCATCTGAGGAACCCCTGCCTTCTGCCCTTCTCCCGATCCGGGATCGCTTTGCAGCTTTGTTTCAGCGGGCGCGCGATGATCAAAACGCAGGTTGTCAAACTGACTACGTCCACGCGGCGATTATAGCCGATCAAATGATGAGCAATGCCTCGGAACTGCGTGGGCTACATGGCGATCTGCATCATGAAAACATCATGTTCTCCAGTCGCGGCTGGCTGGTGATAGATCCCGTCGGTCTGGTCGGTGAAGTGGGCTTTGGCGCCGCCAATATGTTCTACGATCCGGCTGACAGAGACGACCTTTGTCTCGATCCTAGACGCATTGCACAGATGGCGGACGCATTCTCTCGTGCGCTGGACGTCGATCCGCGTCGCCTGCTCGACCAGGCGTACGCTTATGGGTGCCTTTCCGCAGCTTGGAACGCGGATGGAGAAGAGGAGCAACGCGATCTAGCTATCGCGGCCGCGATCAAGCAGGTGCGACAGACGTCATACTAGATATCAAGCGACTTCTCCTATCCCCTGGGAACACATCAATCTCACCGGAGAATATCGCTGGCCAAAGCCTTAGCGTAGGATTCCGCCCCTTCCCGCAAACGACCCCAAACAGGAAACGCAGCTGAAACGGGAAGCTCAACACCCACTGACGCATGGGTTGTTCAGGCAGTACTTCATCAACCAGCAAGGCGGCACTTTCGGCCATCCGCCGCGCCCCACAGCTCGGGCAGAAACCGCGACGCTTACAGCTGAAAGCGACCAGGTGCTCGGCGTGGCAAGACTCGCAGCGAACCCGTAGAAAGCCATGCTCCAGCCGCCCGCATTGGAGAAATTCTTCAAATTCCCGTTGCACATAGCCCGGCAATTCCTTTCCCTGCTCTGCCATAAGCGCAGCGAATGCCGGGTAATACTCGTCAACGATCTGATAGAGAAGGGTTTGCTCGGGTCGGTGGCTCTGGTAACGACCAGTATCCCGATCCCGGCTGGCCGTCCTGGCCGCCACATGAGGCATGTTCCGCGTCCTTGCAATACTGTGTTTACATACAGTCTATCGCTTAGCGGAAAGTTCTTTTACCCTCAGCCGAAATGCCTGCCGTTGCTAGACATTGCCAGCCAGTGCCCGTCACTCCCGTACTAACTGTCACGAACCCCTGCAATAACTGTCACGCCCCCCTGCAATAACTGTCACGAACCCCTGCAATAACTGTCACGCCCCCAAACCTGCAAACCCAGCAGGGGCGGGGGCTGGCGGGGTGTTGGAAAAATCCATCCATGATTATCTAAGAATAATCCACTAGGCGCGGTTATCAGCGCCCTTGTGGGGCGCTGCTGCCCTTGCCCAATATGCCCGGCCAGAGGCCGGATAGCTGGTCTATTCGCTGCGCTAGGCTACACACCGCCCCACCGCTGCGCGGCAGGGGGAAAGGCGGGCAAAGCCCGCTAAACCCCACACCAAACCCCGCAGAAATACGCTGGAGCGCTTTTAGCCGCTTTAGCGGCCTTTCCCCCTACCCGAAGGGTGGGGGCGCGTGTGCAGCCCCGCAGGGCCTGTCTCGGTCGATCATTCAGCCCGGCTCATCCTTCTGGCGTGGCGGCAGACCGAACAAGGCGCGGTCGTGGTCGCGTTCAAGGTACGCATCCATTGCCGCCATGAGCCGATCCTCCGGCCACTCGCTGCTGTTCACCTTGGCCAAAATCATGGCCCCCACCAGCACCTTGCGCCTTGTTTCGTTCTTGCGCTCTTGCTGCTGTTCCCTTGCCCGCACCCGCTGAATTTCGGCATTGATTCGCGCTCGTTGTTCTTCGAGCTTGGCCAGCCGATCCGCCGCCTTGTTGCTCCCCTTAACCATCTTGACACCCCATTGTTAATGTGCTGTCTCGTAGGCTATCATGGAGGCACAGCGGCGGCAATCCCGACCCTACTTTGTAGGGGAGGGCGCACTTACCGGTTTCTCTTCGAGAAACTGGCCTAACGGCCACCCTTCGGGCGGTGCGCTCTCCGAGGGCCATTGCATGGAGCCGAAAAGCAAAAGCAACAGCGAGGCAGCATGGCGATTTATCACCTTACGGCGAAAACCGGCAGCAGGTCGGGCGGCCAATCGGCCAGGGCCAAGGCCGACTACATCCAGCGCGAAGGCAAGTATGCCCGCGACATGGATGAAGTCTTGCACGCCGAATCCGGGCACATGCCGGAGTTCGTCGAGCGGCCCGCCGACTACTGGGATGCTGCCGACCTGTATGAACGCGCCAATGGGCGGCTGTTCAAGGAGGTCGAATTTGCCCTGCCGGTCGAGCTGACCCTCGACCAGCAGAAGGCGCTGGCGTCCGAGTTCGCCCAGCACCTGACCGGTGCCGAGCGCCTGCCGTATACGCTGGCCATCCATGCCGGTGGCGGCGAGAACCCGCACTGCCACCTGATGATCTCCGAGCGGATCAATGACGGCATCGAGCGGCCCGCCGCTCAGTGGTTCAAGCGGTACAACGGCAAGACCCCGGAGAAGGGCGGGGCACAGAAGACCGAAGCGCTCAAGCCCAAGGCATGGCTTGAGCAGACCCGCGAGGCATGGGCCGACCATGCCAACCGGGCATTAGAGCGGGCTGGCCACGACGCCCGCATTGACCACAGAACACTTGAGGCGCAGGGCATCGAGCGCCTGCCCGGTGTTCACCTGGGGCCGAACGTGGTGGAGATGGAAGGCCGGGGCATCCGCACCGACCGGGCAGACGTGGCCCTGAACATCGACACCGCCAACGCCCAGATCATCGACTTACAGGAATACCGGGAGGCAATAGACCATGAACGCAATCGACAGAGTGAAGAAATCCAGAGGCATCAACGAGTTAGCGGAGCAGATCGAACCGCTGGCCCAGAGCATGGCGACACTGGCCGACGAAGCCCGGCAGGTCATGAGCCAGACCCAGCAGGCCAGCGAGGCGCAGGCGGCGGAGTGGCTGAAAGCCCAGCGCCAGACAGGGGCGGCATGGGTGGAGCTGGCCAAAGAGTTGCGGGAGGTAGCCGCCGAGGTGAGCAGCGCCGCGCAGAGCGCCCGGAGCGCGTCGCGGGGGTGGCACTGGAAGCTATGGCTAACCGTGATGCTGGCTTCCATGATGCCTACGGTGGTGCTGCTGATCGCATCGTTGCTCTTGCTCGACCTGACGCCACTGACAACCGAGGACGGCTCGATCTGGCTGCGCTTGGTGGCCCGATGAAGAACGACAGGACTTTGCAGGCCATAGGCCGACAGCTCAAGGCCATGGGCTGTGAGCGCTTCGATATCGGCGTCAGGGACGCCACCACCGGCCAGATGATGAACCGGGAATGGTCAGCCGCCGAAGTGCTCCAGAACACGCCATGGCTCAAGCGGATGAATGCCCAGGGCAATGACGTGTATATCAGGCCCGCCGAGCAGGAGCGGCATGGTCTGGTGCTGGTGGACGACCTCAGCGAGTTTGACCTGGATGACATGAAAGCCGAGGGCCGGGAGCCTGCCCTGGTAGTGGAAACCAGCCCGAAGAACTATCAGGCATGGGTCAAGGTGGCCGACGCCGCAGGCGGTGAACTTCGGGGGCAGATTGCCCGGACGCTGGCCAGCGAGTACGACGCCGACCCGGCCAGCGCCGACAGCCGCCACTATGGCCGCTTGGCGGGCTTCACCAACCGCAAGGACAAGCACACCACCCGCGCCGGTTATCAGCCGTGGGTGCTGCTGCGTGAATCCAAGGGCAAGACCGCCACCGCTGGCCCGGCGCTGGTGCAGCAGGCTGGCCAGCAGATCGAGCAGGCCCAGCGGCAGCAGGAGAAGGCCCGCAGGCTGGCCAGCCTCGAACTGCCCGAGCGGCAGCTTAGCCGCCACCGGCGCACGGCGCTGGACGAGTACCGCAGCGAGATGGCCGGGCTGGTCAAGCGCTTCGGTGATGACCTCAGCAAGTGCGACTTTATCGCCGCGCAGAAGCTGGCCAGCCGGGGCCGCAGTGCCGAGGAAATCGGCAAGGCCATGGCCGAGGCCAGCCCAGCGCTGGCAGAGCGCAAGCCCGGCCACGAAGCGGATTACATCGAGCGCACCGTCAGCAAGGTCATGGGTCTGCCCAGCGTCCAGCTTGCGCGGGCCGAGCTGGCACGGGCACCGGCACCCCGCCAGCGAGGCATGGACAGGGGCGGGCCAGATTTCAGCATGTAGTGCTTGCGTTGGTACTCACGCCTGTTATACTATGAGTACTCACGCACAGAAGGGGGTTTTATGGAATACGAAAAAAGCGCTTCAGGGTCGGTCTACCTGATCAAAAGTGACAAGGGCTATTGGTTGCCCGGTGGCTTTGGTTATACGTCAAACAAGGCCGAGGCTGGCCGCTTTTCAGTCGCTGATATGGCCAGCCTTAACCTTGACGGCTGCACCTTGTCCTTGTTCCGCGAAGACAAGCCTTTCGGCCCCGGCAAGTTTCTCGGTGACTGATATGAAAGACCAAAAGGACAAGCAGACCGGCGACCTGCTGGCCAGCCCTGACGCTGTACGCCAAGCGCGATATGCCGAGCGCATGAAGGCCAAAGGGATGCGTCAGCGCAAGTTCTGGCTGACCGACGACGAATACGAGGCGCTGCGCGAGTGCCTGGAAGAACTCAGAGCGGCGCAGGGCGGGGGTAGTGACCCCGCCAGCGCCTAACCACCAACTGCCTGCAAAGGAGGCAATCAATGGCTACCCATAAGCCTATCAATATTCTGGAGGCGTTCGCAGCAGCGCCGCCACCGCTGGACTACGTTTTGCCCAACATGGTGGCCGGTACGGTCGGGGCGCTGGTGTCGCCCGGTGGTGCCGGTAAATCCATGCTGGCCCTGCAACTGGCCGCACAGATTGCAGGCGGGCCGGATCTGCTGGAGGTGGGCGAACTGCCCACCGGCCCGGTGATCTACCTGCCCGCCGAAGACCCGCCCACCGCCATTCATCACCGCCTGCACGCCCTTGGGGCGCACCTCAGCGCCGAGGAACGGCAAGCCGTGGCTGACGGCCTGCTGATCCAGCCGCTGATCGGCAGCCTGCCCAACATCATGGCCCCGGAGTGGTTCGACGGCCTCAAGCGCGCCGCCGAGGGCCGCCGCCTGATGGTGCTGGACACGCTGCGCCGGTTCCACATCGAGGAAGAAAACGCCAGCGGCCCCATGGCCCAGGTCATCGGTCGCATGGAGGCCATCGCCGCCGATACCGGGTGCTCTATCGTGTTCCTGCACCATGCCAGCAAGGGCGCGGCCATGATGGGCGCAGGCGACCAGCAGCAGGCCAGCCGGGGCAGCTCGGTACTGGTCGATAACATCCGCTGGCAGTCCTACCTGTCGAGCATGACCAGCGCCGAGGCCGAGGAATGGGGTGTGGACGACGACCAGCGCCGGTTCTTCGTCCGCTTCGGTGTGAGCAAGGCCAACTATGGCGCACCGTTCGCTGATCGGTGGTTCAGGCGGCATGACGGCGGGGTGCTCAAGCCCGCCGTGCTGGAGAGGCAGCGCAAGAGCAAGGGGGTGCCCCGTGGTGAAGCCTAAGAACAAGCACAGCCTCAGCCACGTCCGGCACGACCCGGCGCACTGTCTGGCCCCCGGCCTGTTCCGTGCCCTCAAGCGGGGCGAGCGCAAGCGCAGCAAGCTGGACGTGACGTATGACTACGGCGACGGCAAGCGGATCGAGTTCAGCGGCCCGGAGCCGCTGGGCGCTGATGATCTGCGCATCCTGCAAGGGCTGGTGGCCATGGCTGGGCCTAATGGCCTAGTGCTTGGCCCGGAACCCAAGACCGAAGGCGGACGGCAGCTCCGGCTGTTCCTGGAACCCAAGTGGGAGGCCGTCACCGCTGAATGCCATGTGGTCAAAGGTAGCTATCGGGCGCTGGCAAAGGAAATCGGGGCAGAGGTCGATAGTGGTGGGGCGCTCAAGCACATACAGGACTGCATCGAGCGCCTTTGGAAGGTATCCATCATCGCCCAGAATGGCCGCAAGCGGCAGGGGTTTCGGCTGCTGTCGGAGTACGCCAGCGACGAGGCGGACGGGCGCCTGTACGTGGCCCTGAACCCCTTGATCGCGCAGGCCGTCATGGGTGGCGGCCAGCATGTGCGCATCAGCATGGACGAGGTGCGGGCGCTGGACAGCGAAACCGCCCGCCTGCTGCACCAGCGGCTGTGTGGCTGGATCGACCCCGGCAAAACCGGCAAGGCTTCCATAGATACCTTGTGCGGCTATGTCTGGCCGTCAGAGGCCAGTGGTTCGACCATGCGCAAGCGCCGCCAGCGGGTGCGCGAGGCGTTGCCGGAGCTGGTCGCGCTGGGCTGGACGGTAACCGAGTTCGCGGCGGGCAAGTACGACATCACCCGGCCCAAGGCGGCAGGCTGACCCCCCCCACTCTATTGTAAACAAGACATTTTTATCTTTTATATTCAATGGCTTATTTTCCTGCTAATTGGTAATACCATGAAAAATACCATGCTCAGAAAAGGCTTAACAATATTTTGAAAAATTGCCTACTGAGCGCTGCCGCACAGCTCCATAGGCCGCTTTCCTGGCTTTGCTTCCAGATGTATGCTCTTCTGCTCCTGCAGTTCGGGGGCATGGATGCGCGGATAGCCGCTGCTGGTTTCCTGGATGCCGACGGATTTGCACTGCCGGTAGAACTCCGCGAGGTCGTCCAGCCTCAGGCAGCAGCTGAACCAACTCGCGAGGGGATCGAGCCCGGGGTGGGCGAAGAACTCCAGCATGAGATCCCCGCGCTGGAGGATCATCCAGCCGGCGTCCCGGAAAACGATTCCGAAGCCCAACCTTTCATAGAAGGCGGCGGTGGAATCGAAATCTCGTGATGGCAGGTTGGGCGTCGCTTGGTCGGTCATTTCGCCTCGAGGCGAACCCCAGAGTCCCGCTCAGAAGAACTCGTCAAGAAGGCGATAGAAGGCGATGCGCTGCGAATCGGGAGCGGCGATACCGTAAAGCACGAGGAAGCGGTCAGCCCATTCGCCGCCAAGCTCTTCAGCAATATCACGGGTAGCCAACGCTATGTCCTGATAGCGGTCCGCCACACCCAGCCGGCCACAGTCGATGAATCCAGAAAAGCGGCCATTTTCCACCATGATATTCGGCAAGCAGGCATCGCCATGGGTCACGACGAGATCCTCGCCGTCGGGCATGCGCGCCTTGAGCCTGGCGAACAGTTCGGCTGGCGCGAGCCCCTGATGCTCTTCGTCCAGATCATCCTGATCGACAAGACCGGCTTCCATCCGAGTACGTGCTCGCTCGATGCGATGTTTCGCTTGGTGGTCGAATGGGCAGGTAGCCGGATCAAGCGTATGCAGCCGCCGCATTGCATCAGCCATGATGGATACTTTCTCGGCAGGAGCAAGGTGAGATGACAGGAGATCCTGCCCCGGCACTTCGCCCAATAGCAGCCAGTCCCTTCCCGCTTCAGTGACAACGTCGAGCACAGCTGCGCAAGGAACGCCCGTCGTGGCCAGCCACGATAGCCGCGCTGCCTCGTCCTGCAGTTCATTCAGGGCACCGGACAGGTCGGTCTTGACAAAAAGAACCGGGCGCCCCTGCGCTGACAGCCGGAACACGGCGGCATCAGAGCAGCCGATTGTCTGTTGTGCCCAGTCATAGCCGAATAGCCTCTCCACCCAAGCGGCCGGAGAACCTGCGTGCAATCCATCTTGTTCAATCATGCGAAACGATCCTCATCCTGTCTCTTGATCAGATCTTGATCCCCTGCGCCATCAGATCCTTGGCGGCAAGAAAGCCATCCAGTTTACTTTGCAGGGCTTCCCAACCTTACCAGAGGGCGCCCCAGCTGGCAATTCCGGTTCGCTTGCTGTCCATAAAACCGCCCAGTCTAGCTATCGCCATGTAAGCCCACTGCAAGCTACCTGCTTTCTCTTTGCGCTTGCGTTTTCCCTTGTCCAGATAGCCCAGTAGCTGACATTCATCCGGGGTCAGCACCGTTTCTGCGGACTGGCTTTCTACGTGTTCCGCTTCCTTTAGCAGCCCTTGCGCCCTGAGTGCTTGCGGCAGCGTGAA |
| --- |
